# Pharmacological and psychosocial interventions for night eating syndrome in adults

**DOI:** 10.3389/fpsyt.2025.1626342

**Published:** 2025-09-05

**Authors:** Lucas Ribeiro dos Santos, Márcio Luís Duarte, Virgínia Fernandes Moça Trevisani, Maria Stella Peccin, Tamara Melnik

**Affiliations:** ^1^ Department of Evidence-Based Healthcare, Federal University of Sao Paulo, Cochrane Center of Brazil, Sao Paulo, Brazil; ^2^ Department of Medicine, University of Ribeirão Preto, Guarujá, Sao Paulo, Brazil

**Keywords:** obesity, night eating syndrome, food addiction, eating disorder, psychotherapy

## Abstract

**Background:**

Pathological dietary patterns are influenced by various interacting factors, including psychoactive drugs, psychological and biological conditions, and environmental determinants, and are frequently associated with failure in conventional weight loss treatments, especially in obese individuals. Night eating syndrome (NES) is characterized by excessive food consumption at night, often linked to disrupted circadian rhythms and psychosocial triggers.

**Methods:**

This review evaluated pharmacological and psychosocial interventions for NES in adults. Randomized controlled trials (RCTs) comparing psychological or pharmacological interventions versus control groups were included. Primary outcomes were symptom improvement (reduced nighttime eating/awakenings) and weight loss. Secondary outcomes included changes in quality of life, psychiatric comorbidities, sleep quality, interpersonal functioning, and patient satisfaction. We conducted a systematic search in CENTRAL, MEDLINE, EMBASE, Psych INFO, LILACS, ClinicalTrials.gov, and the WHO’s International Clinical Trials Registry Platform.

**Results:**

A total of 5 RCTs were included. Due to heterogeneity in interventions, a meta-analysis was not feasible, and results were presented narratively. Pharmacological interventions trials (Sertraline, Escitalopram, Agomelatine) showed mixed results in reducing NES symptoms, with Sertraline demonstrating the most significant improvements. Psychosocial interventions, including progressive muscle relaxation and education, also showed some benefits, particularly in reducing evening food intake and improving morning hunger. Weight loss outcomes were variable, with some trials showing modest weight loss in intervention groups.

**Conclusion:**

Both pharmacological and psychosocial interventions may provide potential benefits in treating NES. Sertraline shows promise in reducing symptoms and improving quality of life, while psychosocial interventions, particularly progressive muscle relaxation, can modify eating behaviors. However, the heterogeneity of interventions and limited number of studies and subjects included determined a downgraded level of recommendation in GRADE for all outcomes to LOW, suggesting gaps and the need for further research to establish optimal treatment strategies for NES.

## 1 Background

Various interacting factors can contribute to a pathological dietary pattern, including the action of psychoactive drugs (appetite suppressants, psychotropic medications such as antipsychotics, anticonvulsants, and antidepressants, and the action of exogenous hormones), psychological and biological conditions (stress, anxiety, depression, eating disorders, obesity, diabetes), and environmental determinants (foods high in fats and sugars, obesogenic environments, stressful and sedentary lifestyles) (1–3).

Obese individuals with pathological eating profiles often fail to respond to conventional weight loss treatments. The two dietary patterns most frequently associated with treatment failure in weight loss are binge eating disorder (BED) and night eating syndrome (NES) (4–7). Interestingly, the symptoms of these two dysfunctional dietary patterns seem to worsen proportionally with increases in body mass index (8–11).

NES is characterized by different eating behaviors, notably excessive food consumption primarily during the night, with restrictions during the day (6, 12, 13). Food intake occurs in recurrent episodes of binge eating before sleep, often accompanied by insomnia, awakenings to eat, or both. This pattern is frequently associated with the consumption of high-calorie snacks. (6, 12, 13).

NES may be related to a disruption in the normal circadian rhythm, resulting in an inversion of the sleep-wake cycle (14–16). psychosocial and emotional triggers seem to be associated with this dietary pattern (3, 17, 18).

It is known that both biological and psychological factors play important roles in the development and persistence of NES and in the correlation with other psychological disorders, being characterized as a vicious cycle that tends to self-perpetuate (17); moreover, guilt (a common characteristic in eating disorders) regarding night eating pattern, may trigger daytime food restriction and collaborate to maintain maladaptive eating behavior (19). Therefore, it is essential to address the relationship between eating habits, sleep and emotional disorders, breaking this cycle; therefore, combinations of psychological approaches and pharmacotherapy are often used (3, 20).

NES typically occurs in the second and third decades of life, with a slight preference for females—60% of affected individuals (8). The prevalence of NES in the United States is approximately 1.5% of the population, with higher rates of the syndrome associated with greater degrees of obesity - 10% of individuals seeking treatment for obesity, and 27% among candidates for bariatric surgery (20–22).

## 2 Condition description

Diagnostic criteria for NES require recurrent nighttime, of at least 25% of daily caloric intake after dinner and/or waking in the middle of the night to eat, occurring in at least two episodes per week, for at least three months, usually accompanied by the belief that one needs to eat to fall asleep, depression, and morning anorexia (13).

Major differential diagnosis of NES includes Night Eating Disorder (a parasomnia), Binge Eating Disorder and Bulimia Nervosa (6, 8).

## 3 Intervention description

As with other types of eating disorders and obesity, the treatment of NES should address the complex interaction between nutritional, biological, and psychological factors (8, 10, 20, 23, 24).

Regarding pharmacological treatments, anxiolytics and hypnotics, as well as melatonin have been tested. (3, 8, 20, 25, 26).

Anticonvulsants, such as topiramate, are also used for weight loss and psychiatric disorders such as anxiety, binge eating, and depression (11). Additionally, dopaminergic medications such as pramipexole may also be effective in reducing episodes of night eating syndrome (26).

As for psychological interventions, cognitive-behavioral therapy (CBT) is currently the most tested psychotherapy modality. (20, 23, 27–29). Some other behavioral strategies, such as contingency management, stress management, and behavioral interventions for weight loss, have preliminary evidence for reducing the symptoms of night eating syndrome (20).

### 3.1 Importance of conducting this review

The worldwide ‘epidemic’ of obesity, and its association with dysfunctional dietary patterns such as NES, justifies the need to evaluate the efficacy and safety of pharmacological and psychosocial treatments for this subgroup of individuals.

## 4 Objectives

To evaluate the effects of pharmacological and psychosocial interventions on NES in adults.

## 5 Methods

### 5.1 Criteria for considering studies for this review

Types of studies: we included randomized controlled trials (RCTs). We excluded quasi-randomized trials, such as those in which allocation was alternated or sequential, or uncontrolled studies, case series, case reports or other types of primary and secondary studies.

Following Lefevre’s and Cochrane’s guidance, our inclusion criteria were aligned with the PICO framework (Population, Intervention, Comparator, Outcome):

Population: Adults (18 years or older) with a confirmed diagnosis of NES according to diagnostic criteria (6, 13).

To assess the symptoms, various semi-structured interviews and self-reports are available (12, 20, 29, 30):

- Night Eating Questionnaire;
- Night Eating Syndrome History and Inventory;
- Night Eating Syndrome Symptom Scale;
- Eating Disorders Examination (nighttime intakes).

Interventions: any psychosocial or pharmacological interventions evaluated by available trials were considered viable.

Comparator: We included studies comparing psychosocial or pharmacological interventions with control, placebo or no intervention (Waiting list, Placebo, No intervention).

Outcomes:

*Primary Outcomes*: Symptom Improvement, Weight Loss and Adverse Events

*Secondary Outcomes*: Health-Related Quality of Life, Improvement in Psychiatric Comorbidities and Sleep Quality

### 5.2 Search methods for identifying studies

Searches were conducted using Cochrane Highly Sensitive Search Strategy, in the following databases: CENTRAL, MEDLINE, Embase, PsycINFO, LILACS, ClinicalTrials.gov, and WHO ICTRP, with no restrictions on language or year of publication, up to march 2025.

### 5.3 Data collection and analysis

Two reviewers (LRS, TM) independently examined both abstract and title of retrieved records and extracted data from included studies. Both authors also independently assessed the risk of bias for each included study using Cochrane Collaboration’s Risk of Bias 2.0 tool (31). Any discrepancies were resolved by consensus or by consulting a third author (MLD).

We presented a flowchart adapted from Reporting Items for Systematic Reviews and Meta-Analyses (PRISMA) showing the study selection process (32), the overall quality of evidence for each outcome according to the Grading of Recommendations Assessment, Development and Evaluation (GRADE) approach (33), as well as a summary of evidence in a ‘Summary of Results’ table.

## 6 Results

**Figure 1:**
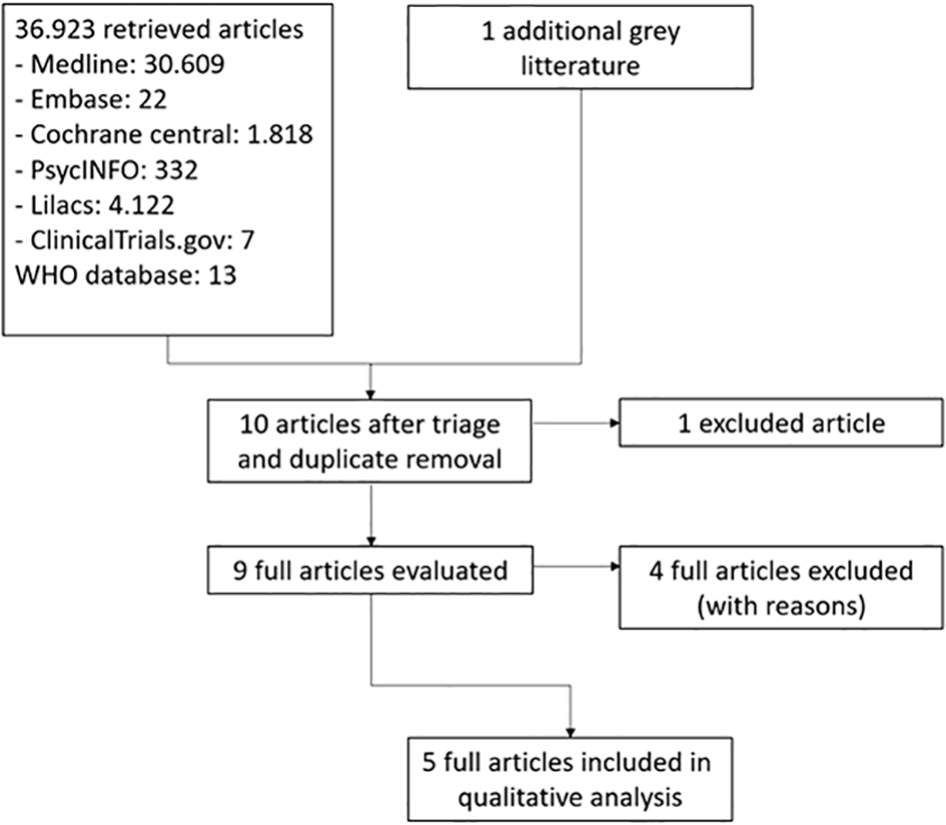
PRISMA.

Our initial search yielded 12,003 studies from the databases and search for alternative sources resulted in one additional study. Following Cochrane collaboration standards for intervention systematic reviews, we excluded non-related studies, narrative reviews, case reports and duplicates, and examined the abstracts of 10 publications - of which 9 had their full texts evaluated, as the Tek (34) protocol found in the clinicaltrials.gov database was withdrawn. The studies by Allison et al. (13) O’Reardon et al. (35), and McCune et al (36) were excluded as they were pre- and post-treatment comparison studies without a control group; the study by Winkelman (37) was excluded as it was a case series. All other 5 studies were included in our review – these results are represented in **Figure 1** – PRISMA flowchart.

Due to the differences in interventions utilized in the included studies, a meta-analysis could not be performed; therefore, we opted to present the results narratively.

### 6.1 Included studies

All five trials that met initial selection criteria had the full text evaluated for inclusion and data extraction using standard form by The Cochrane Collaboration (31).

#### 6.1.1 General characteristics

In total, five studies were included in this review: O’Reardon et al. (38), Vander Wal (39), Vander Wal (40), and Pawlow et al. (41) were published in English with their full texts available, while the study by Makhortova et al. (42) was published as a conference poster. All included studies were conducted in the United States of America, but the average income ranges of the included patients were not reported. The four studies published in full included adult patients (over 18 years of age) with overweight or obesity (mean BMI above 25 kg/m²) - only O’Reardon et al. (38) included 3 patients with normal weight in each group (placebo and sertraline). Makhortova et al. (42) included patients aged 31 to 65 years but did not provide anthropometric data for the sample. The studies by O’Reardon et al. (38), Vander Wal (40), Vander Wal (40), and Pawlow et al. (41) followed an exclusion protocol for patients with parasomnias and decompensated psychiatric disorders or those using psychotropic medication or already enrolled in a weight loss program; such data were not reported in the study by Makhortova et al. (42).

All included studies diagnosed Night Eating Syndrome (NES) using the standardized questionnaire (NEQ – 12), except for the Makhortova et al. (42), which did not provide such information.

#### 6.1.2 Main characteristics of included studies

Main characteristics and results are depicted in **Table 1** – Summary of findings. The interventions and comparators used, as well as the duration of the included studies, varied across them. O’Reardon et al. (38) evaluated 34 patients randomized to Sertraline or placebo, with an initial dose of 50mg, titratable up to 200mg, over 8 weeks; however, the method of participant randomization was not reported. The average daily dose of sertraline at the end of the study was 126.5 mg (SD=50.4). In contrast, the average “dose” of placebo was 173.5 mg (SD=40.0). Vander Wal (40) evaluated, through a randomized double-blind study 1:1 across 2 academic centers over 12 weeks, the efficacy of escitalopram (doses of 10 to 20mg) versus placebo; 40 patients (20 in each group, with equal distribution of men and women, aged 18 to 70 years (mean age of 45 years) with BMI of 25 to 50 kg/m² were included.

**Table 1:** Summary of findings.

Outcome	Trial	Comparative Risks (IC 95%)			Relative effect (IC 95%)	N participants	GRADE	Comments
		Intervention		Control				
NES symptoms improvement	O´Reardon 2005 (38)	Sretralin		Placebo		Sertraline	⊕⊕⊝⊝ Low	Limited number of patients, different interventions, downgrade for imprecision and inconsistency, and some concerns regarding domain 3 in the risk of bias of Pawlow's study, and in D1 of Makhortova, and high risk in D2 of Makhortova
	*CGI*	12/17 score ≤2		3/17 score ≤2	S p<0.001	(N=17)		
	*NEQ*	31.7 ± 5.6→18.1		30.5± 6.2→5.0	I:-57% X P:-16%	Placebo		
	*Night awakening*	8.8 ± 8.6→2.3 ± 4.7		6.4 ± 4.6→5.5 ± 5.0	I:-74% X P:-14%	(N=17)		
	*Night Eating*	8.3 ± 8.5→1.6 ± 2.6		6.4 ± 4.9→5.5 ± 4.9	I:-81% X P:-14%			
	Vander Wal 2012 (39)	Escitalopram		Placebo		Escitalopram		
	*NEQ*	-13.0 ( ± 1.60)		-10.6 ( ± 2.2)	- 2.40 ( ± 10.3)	(N=20)		
	*CGI*	12/17 score ≤2		3/17 score ≤2	NS P =0.113	Placebo		
	*NESHI* *(respondedors)*	16 (80%)		12 (60%)	NS p=0.168	(N=20)		
	Vander Wal 2015 (40)	PMR	PMR plus	E		PMR (N=38)		
	*NEQ*	−7.16 ± 6.64	−8.38± 7.43	−9.13 ± 11.00	NS p=0,07	PMR plus E		
	*NEDQ*					(N=38)		
	Sleep hours/night	0.69 ± 2.11	0.53 ± 1.12	0.23 ± 0.81	All groups significantly reduced NEQ scores, but with no statistically significant difference between groups	E (N=38)		
	Breakfast N+week	2.50 ± 2.25	0.75 ± 0.35	1.06 ± 2.56				
	%eating after 19h	-7.54 ± 23.94	-30.54 ± 29.26	-22.85 ± 14.71				
	%eating after dinner	-30.54 ± 19.84	-20.42 ± 15.56	-9.50 ± 23.52				
	N initial insomnia	0.00 ± 0.50	-0.67 ± 1.15	-0.75 ± 2.10				
	N maintenance insomnia	1.62 ± 5.18	1.89 ± 8.01	3.06 ± 5.94				
	N awakenings/week	-1.86 ± 2.63	-3.00 ± 5.17	-3.90 ± 10.76				
	Pawlow et al., 2003 (41)					APRT (N=10)		
	*NEQ*	Not described		Not described	APRT: morning hunger higher (P < 0.05) and lower at 9:00 p.m. (P < 0.025). Nearly significant trends for > N breakfasts (P = 0.08) and < N awakenings to eat after bedtime (P = 0.06)	Control (N=10)		
	Makhortova 2014 (42)	*Eating before bed*		*Feeding after bed*		Feeding before bedtime (N 57)		
		Agomelatina	Sertralina	Agomelatina	Significant reductions (p<0.05) in both groups, with a greater reduction in the Agomelatine group for those waking to eat, and greater in the Sertraline group for those eating before bedtime	Alimentação após deitar (N 39) N was not described for each group Sertraline and Agomelatine		
	*NEQ*	27,00 ± 1,75	22,28 ± 1,08	23,16 ± 1,32				
Weight loss	O´Reardon 2005 (38)	- 2.9 kg ( ± 3.8)		-0.3 kg ( ± 2.7)	2.60 [0.16, 5.04], p=0,06	Sertraline (N=14) Placebo (N=14) *Only overweight patients included*	⊕⊕⊝⊝ Low	Limited number of patients, different interventions, downgrade due to imprecision and inconsistency
	Vander Wal 2012 (39)	-0.43kg (0.7)		+1.12 kg (0.6)	-1.55 [-1.95, -1.15], p= 0.086	Escitalopram (N=20) Placebo (N=20)		
	Pawlow et al., 2003 (41)	-0.81 kg		+0.27 kg)	P=0,07	APRT (N=10) Controle (N=10)		
Adverse events	O´Reardon 2005 (38)				Side effects were mild and included dry mouth, fatigue, decreased libido, and sweating. Nausea as an adverse event was infrequent and transient (n=2 placebo and n=1 sertraline).	Sertraline (N=17) Placebo (N=17)	⊕⊕⊝⊝ Low	Limited number of patients, different interventions, downgrade due to imprecision and inconsistency
		Not described		Not described				
	Vander Wal 2012 (39)	Headache (n=5), Gastrointestinal symptoms (n=4), Upper respiratory infections/seasonal allergies (n=3), Fatigue (n=3), Sexual problems (n=2) Cognitive symptoms (n=2) Drowsiness (n=4),		Headache (n=2) Gastrointestinal (n=2) Upper respiratory infections/seasonal allergies (n=3),	“none of the adverse events occurred more frequently in the escitalopram group than in the placebo group (P values of 0.231 to 0.487)”	Escitalopram (N=20) Placebo (N=20)		
Health-related quality of life	O´Reardon 2005 (38)	54.3pts ( ± 9.6)		47.4pts ( ± 7.3)	6.90 [1.17, 12.63], p=0,045	Sertraline (N=17)	⊕⊕⊝⊝ Low	Limited number of patients, different interventions, downgrade due to imprecision and inconsistency
	[Quality of Life Pleasure and Satisfaction Questionnaire]					Placebo (N=17)		
Improvement in psychiatric comorbidities	O´Reardon 2005 (38)					Sertraline (N=17) Placebo (N=17)	⊕⊕⊝⊝ Low	Limited number of patients, different interventions, downgrade due to imprecision and inconsistency
	*BDI*	14.4 ( ± 9.7) →?		12.1 ( ± 9.5) →?	BDI score change p=0.10			
	*EDH*	9.9 ( ± 4.5) )→?		9.6 ( ± 5.2) )→?	EDH score change p=0.20			
	Vander Wal 2012 (39)					Escitalopram (N=20) Placebo (N=20)		
	*BDI*	-2.4 (1.2)		-3.5 (1.5)	1.10 [0.26, 1.94] p=0.595			
	*BAI*	-1.5 (0.8)		-1.8 (0.9)	0.30 [-0.36, 0.96] P=0.420			
	Vander Wal 2015 (40)	PMR	PMR plus	E		PMR (N=38) PMR plus E (N=38) E (N=38)		
	*BDI*	-7.38 ( ± 6.42)	-2.85 ( ± 8.79)	0.50 ( ± 11.20)	P < 0,05			
	*BAI*	-2.92 ( ± 4.80)	-1.08 ( ± 1.47)	-6.25 ( ± 9.12)	NS			
	*PSS*	-3.54 ( ± 7.20)	-3.16 ( ± 6.88)	-1.92 ( ± 11.24)	NS			
	Pawlow et al., 2003 (41)					APRT (N=10) Controle (N=10)		
	*STAI*	32.9 ( ± 14.6)		48.4 ( ± 15.3)	P < 0,05 P< 0,05 P< 0,05 * P<0,05 Pre→post * P<0,05 Pre→post ^¥^p<0.05 vs Control.			
	*RRS*	7.4 ( ± 2.1)		3.7 ( ± 2.1)				
	*PSS*	26.8 ( ± 4.9)		31.6 ( ± 6.3)				
	*BDI*	15.9 ( ± 11.3)→8.2 ( ± 10.3)^¥^		9.4 ( ± 6.4) → 9.9 ( ± 6.5)*				
	*POMS Anger*	51.8 ( ± 9.2) →42.8 ( ± 10.0) ^¥^		43.1 ( ± 4.5) →44.4 ( ± 5.3)*				
	*POMS Depression*	45.0 ( ± 7.6) →36.9 ( ± 7.6)*		39.0 ( ± 4.1) →40.9 ( ± 6.1)*				
	*POMS Fatigue*	49.0 ( ± 7.9) →40.3 ( ± 7.4)*		46.7 ( ± 6.4) →46.2 ( ± 5.3)*				
	*POMS Tension*	44.4 ( ± 6.4) →44.2 ( ± 6.6)		40.9 ( ± 7.8) →42.0 ( ± 6.7)				
	*POMS Vigor*	54.4 ( ± 8.9) →58.3 ( ± 13.7)		65.2 ( ± 10.8)→62.5 ( ± 10.5)				
	*POMS Confusion*	43.2 ( ± 5.9) →41.4 ( ± 6.0)		39.9 ( ± 5.7) →41.2 ( ± 7.2)				
Sleep improvement	Vander Wal 2015 (40)	PMR	PMR plus	E		Variable	⊕⊕⊝⊝ Low	Limited number of patients, downgrade due to inaccuracy and inconsistency
	*Sleep latency (min)*	2.24 ( ± 22.02)	-6.39 ( ± 12.45)	1.37 ( ± 48.77)		19		
	*N° awakenings/week*	-1.59 ( ± 3.56)	-4.09 ( ± 2.98)	-1.78 ( ± 12.38)		31		
	*Sleep quality*	0.88 ( ± 1.20)	0.35 ( ± 0.90)	0.33 ( ± 0.69)		31		
	*Feeling rested*	0.58 ( ± 1.27)	0.15 ( ± 0.71)	0.28 ( ± 0.66)		30		

GRADE Working Group Grades of Evidence.

High quality: Future research is unlikely to affect our confidence in the effect estimate.

Moderate quality: Future research is likely to have an impact and may affect our confidence in the effect estimate.

Low quality: Future research is likely to have an important impact on our confidence and may change the effect estimate.

Very low quality: We are uncertain about the estimate.

The studies by Vander Wal (40) and Pawlow et al. (41) were psychotherapy intervention studies. Vander Wal (40) compared the effects of Education, progressive muscle relaxation therapy (PMR), and exercise over 3 weeks, dividing them into 3 groups: PMR, PMR+Exercise, and Education; in the Education group, patients received an educational presentation via PowerPoint and discussion on NES, healthy eating, and the importance of sleep hygiene, along with informational materials for note-taking and sleep recording at home. The PMR group received the same educational presentation as the Education group, with the addition of a section on using PMR in the treatment of NES; PMR+Exercise group engaged in the same activities as the PMR group, with the addition of a section on the role of exercise in treating NES.

The trial by Pawlow et al. (41) evaluated the impact of the abbreviated progressive muscle relaxation technique (APRT) on NES in 20 patients (19 women and 1 man, allocated to the experimental group) with a mean age of 38 years (+/- 10.5) and a BMI of 34kg/m² (+/- 11) randomized between control and experimental groups. The intervention group underwent a standardized APRT session for 20 minutes in a low-light room, while the control group simply sat in a lit room; patients were instructed to repeat these sessions daily before bedtime. All scales were administered before and after the first session and before the eighth day (end of the trial); during the study week, an average of 5.8 home sessions were completed.

The study by Makhortova et al. (42), published as a poster, evaluated patients with NES, dividing them between a group that consumed most of their food post-dinner but before bed, and a second group that ate after lying down, leading to nighttime awakenings; each group was then divided and randomized to receive Sertraline 50-100mg/day or agomelatine (a potent melatonin receptor agonist and serotonin-2C receptor antagonist) 25-50mg/day. The groups were assessed for NEQ scores before the intervention and after 60 days.

#### 6.1.3 Risk of bias in included studies

**Figure 2:**
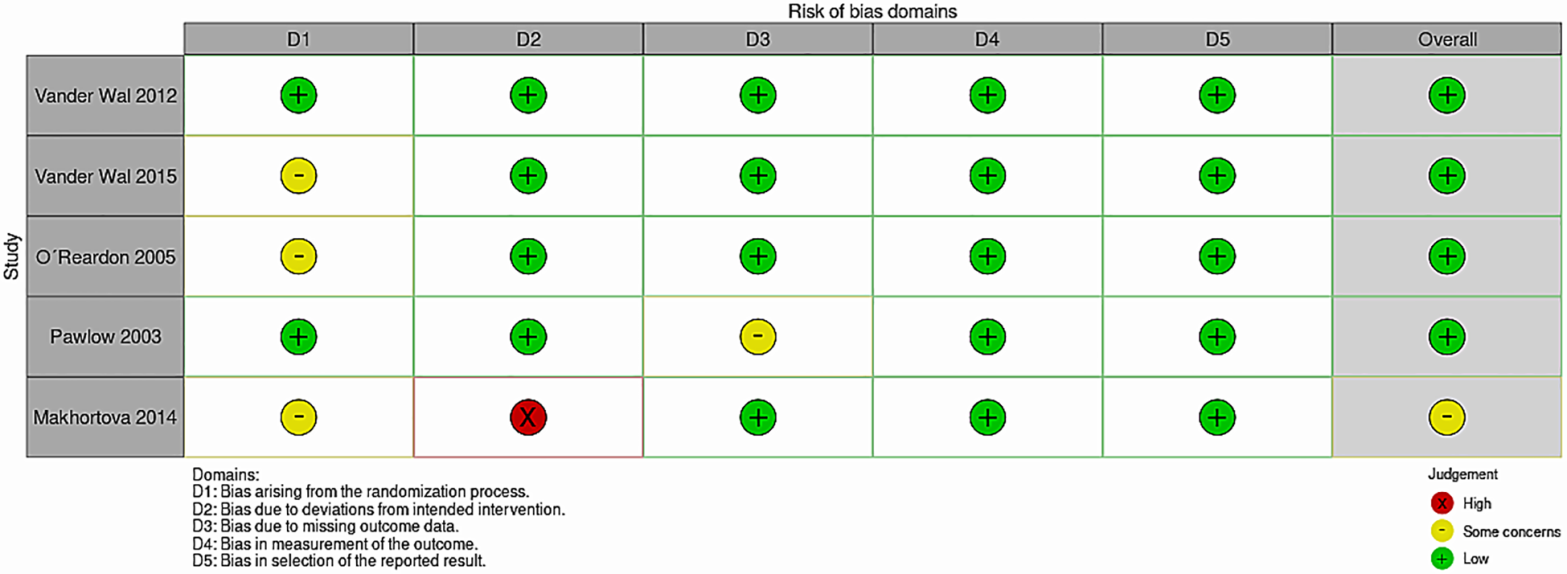
Risk of bias table 2.0.

The overall risk of bias was considered low for most studies, except for the Makhortova et al. (42) (as it was a panel from congress presentation, that usually presents limitations in some methodological descriptions – which yielded more concerns in risk of bias judgments), with specific domains for each trial described in **Figure 2**, according to the Cochrane collaboration Risk of Bias 2 (RoB 2) tool.

The studies by Vander Wal (40) and O’Reardon et al. (38) did not report the method of randomization; the study by Pawlow et al. (41) did not report the score of a primary outcome (NES questionnaire) for each group, only reporting the mean score for the overall sample and stating that there were no significant differences between groups.

### 6.2 Primary outcomes

#### 6.2.1 Symptom improvement

The study by O’Reardon et al. (38) observed, through the CGI (Clinical Global Impression) score, that 12 of the 17 patients in the sertraline group were responders (score ≤2), with 7 of these 12 achieving remission or complete resolution (F=6.7, df=4, 113, p<0.001). Among those receiving placebo, only 3 individuals were classified as responders, with 1 complete remission (response rate significantly lower than sertraline [χ2 = 9.66, df=1, p<0.002]). Of the three patients with normal weights in the sertraline group, two responded, while none of the 3 in the placebo group achieved a response rate. Interestingly, the greatest reduction in symptoms occurred by week 2, with a 30% chance of response in the first 15 days (with 5 responders and 4 in remission status); on the other hand, the lack of early improvement did not exclude the final response, as 50% of all responses occurred between weeks 4 and 8. The sertraline group had a two-point reduction on the CGI severity scale (from 4.2 - moderate severity - to 2.2 at week 8 - borderline, with a much more modest result (from 4.2 to 3.4) in the placebo group (F=4.1, df=4, 107, p=0.004).

As for the nighttime eating symptoms score, at week 8, the sertraline group exhibited a decrease of 18.1 points (57%) compared to baseline (31.7 points), versus a reduction of only 5 points (16%) in the placebo group (F=8.0, df=4, 112, p<0.0001). There was a significant correlation between the rate of decrease in score in the first 2 weeks and the change from baseline to week 8 in the sertraline group (r=0.68, p=0.01), indicating that early improvement with sertraline was predictive of the final response. Additionally, those who responded early showed improvement with lower doses compared to those who responded later (r=0.84, p<0.001), although sertraline dose was not an isolated predictor of responses.

When evaluating episodes of awakening and nighttime eating, the number of nighttime intakes in the sertraline group fell by 81% (initial average of 8.3 episodes/week [SD=8.5] to 1.6 [SD=2.6]) versus a decrease of only 14% in the placebo group (from 6.4 [SD=4.9] to 5.5 [SD=4.9] per week) (F=3.7, df=4, 80, p=0.01). The number of awakenings fell by 74% in the sertraline group (from an average of 8.8 per week [SD=8.6] to 2.3 [SD=4.7]) versus a decrease of only 14% in the placebo group (from 6.4 [SD=4.6] to 5.5 [SD=5.0]); however, this decrease did not reach statistical significance in the overall interaction effect (F=0.9, df=4, 80, p=0.40), but produced a difference in the main effect between groups (F=4.7, df=1, 32, p=0.03).

Regarding caloric intake after dinner, there was a decrease of 68% in the sertraline group (from 47.3% of the daily total at baseline to 14.8% at week 8) versus a decrease of 29.3% (from 44.7% to 31.6% at week 8) in the placebo group (F=3.5, df=4, 106, p=0.009).

The study by Vander Wal (40) showed that, when assessing NES symptoms, there was no significant difference in mean change between the Escitalopram and placebo groups (-13.0 [1.6] for escitalopram, and -10.6 [2.2] for placebo, F1,37 = 2.5, P = 0.124). Seven (35%) in the escitalopram group demonstrated a decrease of at least 50% in their NEQ scores compared to 6 (30%) in the placebo group (X2 = 0.11, P = 0.736). Individual item analysis indicated that patients in the escitalopram group showed more hunger in the morning (p = 0.020), less likelihood of needing to eat to return to sleep when waking at night (p = 0.022), and snacking less upon waking during the night (p = 0.031).

In the Clinical Global Impression Improvement Inventory (CGI-I), 12 (60%) in the escitalopram group were classified as having responded to treatment (score < 2), compared to 7 (35%) of the placebo group (X2 = 2.5, P = 0.113). In the evaluation of the NES Inventory and History, 16 (80%) in the escitalopram group versus 12 (60%) in the placebo group no longer met diagnostic criteria (X2 = 1.9, P = 0.168).

In the study by Makhortova et al. (42), the overall NEQ score was significantly higher in the group that awakened to eat (N 39, NEQ Sertraline 38.33 ± 0.89 and NEQ Agomelatine 37.68 ± 1.44, versus NEQ Sertraline 33.97 ± 0.95 and NEQ Agomelatine 32.80 ± 0.80). After 60 days of intervention, it was observed that the NEQ score of patients who ate before bed was significantly lower in the Sertraline group than in the Agomelatine group (22.28 ± 1.08 versus 27.00 ± 1.75). Conversely, in patients who had their meals after lying down, those randomized to receive Agomelatine showed lower scores (30.61 ± 1.09 versus 23.16 ± 1.32).

Vander Wal (40) showed, when evaluating the NES symptom questionnaire, 3 patients in the Education group, 5 in the PMR group, and 4 participants in the PMR Plus group achieved remission (12 out of 38 - 31.6%); moreover, no patients in the Education group, 1 in the PMR group, and 2 in the PMR Plus group showed improvement (3 out of 38 - 7.8%), for a total of 15 out of 38 (39.5%). The difference in improvement between groups was not statistically significant (X2 = 2.84, p = 0.585). In assessing NEQ scores, although all three groups showed statistically significant results in reductions in NEQ scores, there were no statistically significant differences in reductions between groups.

There was an improvement in the number of days consuming breakfast, the proportion of food consumed after 7 PM, the amount of food ingested after dinner, and the number of times getting out of bed per week. The only significant difference between groups was the percentage of food ingested after dinner, with the PMR group showing the largest reduction (-30.54%), followed by the PMR Plus group (-20.42%) and the Education group (-9.5%) – PMR showed a significantly greater decrease than the Education group (p = 0.012); other differences were not statistically significant.

The study by Pawlow et al. (41) did not report NEQ scores as described in its methods but indicated that the APRT group had significantly higher morning hunger scores (t(18) = 2.27, P <0.05) and significantly lower scores at 9 PM (t(18) = 2.83, P<0.025). There were nearly significant trends for a greater number of breakfasts for the APRT group (P = 0.08) and a lower number of awakenings for eating after lying down (M = 0.8) compared to the Controls (M=2.2, P=0.06). In the experimental group, the use of the APRT tape before bedtime was strongly related to nighttime eating: Experimentals reported nighttime eating on only 2.8% of the nights they listened to the tape, compared to 50% of the nights they did not listen to the tape.

In this trial, during the week, for the entire sample, the frequency of nighttime eating episodes was inversely related to average morning hunger ratings (r(17) = 0.508, P<0.05) and the number of breakfasts consumed (r(18) = 0.481, P<0.05), with this last measure being highly correlated (r(17) = 0.853, P<0.001).

#### 6.2.2 Weight loss

O’Reardon et al. (38) reported that, among overweight individuals (N=14 in both groups), the sertraline group lost 2.9 kg (SD=3.8) versus 0.3 kg (SD=2.7) in the placebo group (F=2.6, df=4, 63, p=0.06), achieving a significant difference by week 8 (t=–2.7, df=63, p=0.009): among the 3 individuals with normal weight receiving sertraline, there was a decrease of 1.2 kg compared to a gain of 0.3 kg by the three individuals with normal weight who received placebo.

In the study by Vander Wal (40), BMI was significantly associated with NEQ score changes, with higher BMI associated with greater reductions in NEQ scores (r=0.34, P=0.034); however, there was no significant interaction effect between BMI and treatment group. Regarding race, a trend for differential response was detected (r=0.29, P=0.073); results from a 2x2 ANOVA on NEQ scores showed a marginally significant interaction (P=0.052), with white patients demonstrating a significant response (P=0.024), but black patients did not (P=0.453). There was a slight decrease in body weight for the escitalopram group (-0.43 [0.7] kg) and a slight increase for the placebo group (+1.1 [0.6] kg), although not significant (P=0.086); there were no significant differences between groups regarding changes in blood glucose and lipid profile.

Pawlow et al. (41) showed a marginally significant trend for the Experimental group of -0.81 kg, versus an increase in the control group +0.27 kg (P=0.07). Seven of the 10 participants in the experimental group lost at least 0.45kg during the study, compared to only one from the control group (P<0.025). For the entire sample, the NEQ score was positively correlated with anxiety scores (r(18) = 0.551, P<0.025), but not with BMI (r(18)=0.080, P>0.05) or weight (r(18)=0.226, P>0.05). Weight was positively correlated with many baseline mood indices: BDI (r(18) = 0.524, P<0.025), Anger (r(18) = 0.612, P<0.01), Depression (r(18) = 0.571, P<0.01), Tension (r(18) = 0.713, P<0.001), and Confusion (r(18)=0.556, P<0.025). BMI was also significantly related to various mood measures: BDI (r(18) = 0.460, P<0.05), Anger (r(18) = 0.493, P<0.05), Depression (r(18) = 0.451, P<0.05), and Tension (r(18)=0.640, P<0.01).

#### 6.2.3 Adverse events

O’Reardon et al. (38) described sertraline as well tolerated, and there were no dropouts due to adverse events; the most common side effects were mild and included dry mouth, fatigue, decreased libido, and sweating.

In the Vander Wal (39) trial, patients began the study with 10mg of Escitalopram (or identical placebo) and could be uptitrated in case of inadequate response; in the escitalopram group, 3 patients were unable to tolerate the maximum dose (all 3 cited drowsiness - 1 cited erectile dysfunction and difficulties concentrating, and were thus maintained on 10 mg). There was one case of discontinuation after week 8 due to fatigue. In the placebo group, dose was reduced for 1 patient due to complaints of drowsiness, dry eyes, and headache.

### 6.3 Secondary outcomes

#### 6.3.1 Health-related quality of life

Only O’Reardon et al. (38) evaluated this outcome, utilizing the Quality of Life Pleasure and Satisfaction Questionnaire. In the sertraline group, there was an increase in the score, from 47.1 (SD=12.0) at baseline to 54.3 (SD=9.6) at week 8, in contrast to the placebo group, which remained essentially unchanged (mean = 47.6 [SD = 9.9] at baseline and 47.4 [SD = 7.3] at week 8; F=2.5, df=4, 108, p=0.045).

#### 6.3.2 Improvement in psychiatric comorbidities

O’Reardon et al. (38) assessed the Hamilton and Beck scales for depressive symptoms (BDI), while Vander Wal (40) employed Beck’s anxiety and depression scales (BAI and BDI). Vander Wal (40) evaluated the effects on the Beck Anxiety Inventory (BAI) and the Beck Depression Inventory (BDI), in addition to the Perceived Stress Scale (PSS), while Pawlow et al. (41) used the State-Trait Anxiety Inventory (STAI), the Relaxation Scale (RRS), the Perceived Stress Scale (PSS), the Beck Depression Inventory (BDI), and the Profile of Mood States (POMS).

##### 6.3.2.1 Anxiety symptoms

In Vander Wal (40), most (n = 33) patients exhibited minimal anxiety according to the BAI (scores of 0 to 7), with 5 presenting mild scores (scores of 8 to 15) and 2 reporting moderate anxiety (scores of 16 to 25). At the end, there were no significant differences between groups concerning changes in anxiety symptoms (P=0.420); however, there was a significant correlation between the change in Beck Anxiety Inventory scores and the change in NEQ scores (P=0.043) for the overall sample.

In the study by Vander Wal (40), the general population exhibited mild scores on the BAI (11.13 ± 8.47 points); all three groups showed statistically significant reductions in BAI scores (final score 7.79 ± 5.28), but no statistically significant difference between groups. Reductions in NEQ scores were not associated with reductions in BAI scores (p=0.113).

##### 6.3.2.2 Depressive symptoms and mood

In O’Reardon et al. (38), mood measures showed only a modest level of depressive symptoms in both groups at baseline, which did not differ over time (change in Hamilton score: F = 1.5, df = 4, 110, p=0.20; Change in Beck score: F=1.9, df=4, 100, p=0.10). There was no significant correlation between changes in NES symptoms and reductions in depressive symptoms assessed by the Beck Inventory or the Hamilton Depression Scale, so the selective removal of the two depression items from the nighttime eating symptom scale showed that changes in depression symptoms were not the main driver of changes in Night Eating Syndrome symptoms.

Similarly, in Vander Wal (40), most patients (n = 32) had minimal depressive symptoms (BDI scores between 0 and 13), with 4 patients endorsing mild scores (scores of 14 to 19) and 4 moderate scores (scores of 20 to 28). At the end, there were no significant differences between groups regarding changes in depressive symptoms (P=0.595).

In the study by Vander Wal (40), the general population exhibited mild scores on the BDI (overall mean 15.76 ± 9.77 points). All three groups showed statistically significant reductions in BDI scores (final score 12.42 ± 10.71), but not statistically significant. The results showed that reductions in NEQ scores were associated with reductions in BDI scores (p=0.003).

In the study by Pawlow et al. (41), except for the Experimental group on Day 1, whose average score on the BDI would be diagnostically classified as “Mildly Depressed,” the scores for both groups were below the threshold for a depression diagnosis at each session. When assessing the response on the BDI, there was a significant effect for Time (F (1,18) = 12.87, P<0.01) which was qualified by a significant interaction of Group X Time (F (1,18) = 16.69, P<0.01), but there was no significant interaction effect between groups (P>0.05). Simple effects tests revealed that the experimental group was significantly more depressed than the controls on Day 1 (F (1,18) = 5.13, P<0.05), but not on Day 8, and there was a significant decrease in depression from Day 1 to Day 8 for the Experimental group (F (1,18) = 7.19, P<0.025), but not for the Control group.

In evaluating mood states, there were significant effects for time, qualified by significant Group x Day interactions, in the domains of Anger (Time F (1,18) = 6.96, P<0.025); Day X Group (F(1,18) = 12.46, P<0.01)), Depression (Time F (1,18) = 6.66, P<0.025); Group X Day F (1,18)=17.31, P<0.01)) and Fatigue (Time F (1,18) = 9.78, P<0.01; Day X Group F(1,18)=7.72, P<0.025)). There were no significant main effects for Group (P>0.05). Simple effects tests showed that the Experimental group scored significantly higher in Anger than the Controls on Day 1 (F (1,18) = 4.68, P<0.05), but not on Day 8; furthermore, from Day 1 to Day 8, there was a significant decrease in anger (F (1,18) = 4.79, P<0.05), Depression (F (1,18) = 4.48, P<0.05) and Fatigue (F(1,18) = 4.51, P<0.05) for the Experimental group, but not for the Controls. There were no significant main effects or interactions on the POMS subscales of Tension, Vigor, or Confusion (P>0.05).

##### 6.3.2.3 Stress symptoms

The PSS is a self-report measure of 14 items that assesses the degree to which events in someone’s life are perceived as stressful, with scores ranging from 0 to 56 points – In the study by Vander Wal (40), the general population showed moderate to high levels (27.58 ± 6.94) of stress. All three groups showed statistically significant reductions in PSS scores (final score 24.68 ± 7.91), but not statistically significant; results showed that reductions in NEQ scores were associated with reductions in PSS scores (p = 0.021).

In the study by Pawlow et al. (41), at the end of the study, there was a significant reduction in STAI scores both in the post-session evaluation on Day 1 (F (1,36)=15.90, P<0.01), and in the pre-session evaluation on Day 8 (F (1,36)=12.06, P<0.01); in the experimental group, there was also a gradual reduction over the week (F(2,36)=11.62, P<0.01), not observed in the control group.

Simple effects tests revealed that the relaxation scale (RSS) scores of patients in the experimental group were significantly higher than those of the Controls on Day 1 post-session (F (1,36) = 12.98, P<0.01) and on Day 8 pre-session (F(1,36)=12.53, P<0.01). There were also significant increases over time in the Experimental group (F(2,36) = 10.17, P<0.01), but not in the Control group.

Regarding perceived stress (PSS), a significant interaction of Group X Time was observed (F (2,36) = 5.20, P<0.025), with significantly lower scores in the experimental group on Day 1 post-session (F (1,36) = 4.69,P<0.05) and on Day 8 session (F (1,36)=8.19, P<0.01; there were also significant reductions over time only in the Experimental Group (F (2,36) = 6.89, P<0.01).

After the control individuals received APRT, they underwent the same evaluations in the post-session on Day 8, in order to replicate the effect evaluations within the session performed with the Experimental group on Day 1. Except for salivary cortisol, the significant changes pre and post-APRT found in the Experimental group on Day 1 were replicated on Day 8 for the Control group when they received APRT, showing a significant reduction in their Pre X Post-APRT scores on STAI scores (t (9) = 4.28, P<0.01) and PSS (t (9) = 4.13, P<0.01), and significantly increased in RSS (t (9) = 5.08, P<0.01); however, the significant reduction of salivary cortisol from pre to post-APRT observed in the Experimental group on Day 1 was not replicated for the Controls on Day 8 (t (9) = 1.66, P>0.05), although a similar trend was observed.

Average cortisol levels were above expected for healthy adults during the early morning hours, except for the Experimental group in the post-session on Day 1 (5.22–23.43 nmol/l). The results revealed a main effect between Groups (F (1,18) = 9.40, P<0.01), qualified by a significant interaction effect of Time X Group (F(2,36) = 4.21, P<0.075). A significant decrease in salivary cortisol level from pre to post-session on Day 1 was observed for the Experimental group (F (2,36) = 3.27, P<0.05).

#### 6.3.3 Sleep quality

Only Vander Wal (40) assessed sleep quality, showing significant improvements in the number of hours slept. The analysis of sleep records showed significant improvements in nighttime activities regarding eating episodes and sleep quality, with positive trends for reductions in the number of awakenings per week (p = 0.054) and feeling rested (p = 0.06). However, the overall quality of sleep was still rated as fair, and the sense of being rested was rated as slightly rested, with no significant change in sleep latency. There were no significant differences between groups.

## 7 Discussion

### 7.1 Summary of main results

Our initial search provided a high number of studies; however, most of them were non-structured narrative reviews or case reports. In our attempt to describe the highest available quality of evidence, we performed a systematic review following Cochrane collaboration to meet rigorous methodological standards in addressing the proposed clinical question, but remain useful to the scientific community, even in the face of literature constraints.

All included studies were heterogeneous and differed in interventions; thus, it was not possible to compile the data into a meta-analysis, and we opted for a descriptive analysis of their results. However, all studies adhered to the diagnostic criteria established in the literature (6, 13), making samples somewhat homogeneous and facilitating the analysis of their results; a common issue was the very small sample size, with an N ranging from 10 to 57 patients per intervention group.

The risk of bias in the included studies was considered low in most domains of the included studies, with only a lack of information regarding the randomization process in the studies by Vander Wal (40), Makhortova et al. (42) and O’Reardon et al. (38), and concerns related to domain 3 in the study by Pawlow et al. (41), which did not report the score of a primary outcome (NES questionnaire) for each group, only reporting the mean score of the overall sample and stating that there were no significant differences between groups.

The study by O’Reardon et al. (38) evaluated the use of the anxiolytic/antidepressant Sertraline in treating NES and found a positive result in managing this syndrome, with reductions in the CGI scale, as well as in episodes of nighttime awakening and nighttime eating, and even in quality of life scores and modest weight loss. Interestingly, in the study sample, the depressive symptoms assessed by the Beck and Hamilton inventories did not show a positive correlation between the severity of depression and NES symptoms. Furthermore, there was good tolerance to the medication, as there were no serious adverse effects or dropout cases from the study. In the study by Makhortova et al. (42), sertraline also showed efficacy in reducing the NEQ score (as well as agomelatine, with differences in response based on eating patterns). As so, Makhortova et al. (42) also revealed an improvement in NEQ score with sertraline

In contrast, the study by Vander Wal (40), which also evaluated a serotonin reuptake inhibitor, Escitalopram, found no benefit from this medication, with results comparable to placebo when assessing symptom reduction on the NES questionnaire or the CGI, and there was no significant weight difference at the end of the study. However, in the individual item analysis of the NEQ, patients in the escitalopram group showed more hunger in the morning and were less likely to need to eat to return to sleep. In this study, there was also no difference in the improvement of Beck´s depression and anxiety scores between the escitalopram and placebo groups, but there was a significant correlation between the change in the Beck Anxiety Inventory score and the change in the NEQ score. Moreover, tolerance to medication was moderate, with some side effects preventing dose escalation, with one dropout due to significant fatigue.

It is important to note that, depression and anxiety scores revealed only mild levels of these comorbidities in the population sample at baseline, but even lower in the study by Vander Wal (mean BDI score of 7.2 points, versus 13.3 in the study by O’Reardon); conversely, while in the study by O’Reardon there was no correlation of these psychiatric comorbidities with NES symptoms, such a positive correlation occurred in the study by Vander Wal (40), even presenting minimal symptoms of anxiety and depression.

Recent evidence suggests that sertraline may be more advantageous in terms of weight control (43), as well as more effective in managing moderate to severe depressive symptoms (44, 45), which could explain the discrepancy in results.

Following the reasoning of the association between anxious and depressive symptoms with NES, the studies by Vander Wal (40) and Pawlow et al. (41) assessed the response of relaxation therapies in controlling NES. In the study by Vander Wal (40), both PMR alone and when combined with physical exercise and simple Education toward symptoms showed improvement in controlling NES, as well as in anxiety, depression, and stress scores, with a positive correlation between improvement in the NES score and psychological scores, although no differences were found between the interventions.

In the study by Pawlow et al. (41), although the differences in NEQ scores between groups were not reported, only a reduction in the number of meals post-dinner and increases at breakfast were observed, this trial revealed the same positive association between anxiety, depression, and mood scores. Indeed, the improvement determined by APRT on mood profiles was positively correlated with improvement in eating patterns.

An approach that was not adequately addressed in the studies was the management of insomnia, with only the study by Makhortova et al. (42) evaluating a sleep-inducing medication - agomelatine; we have strong evidence showing a positive association between insomnia disorders and NES (46), and knowing the positive feedback loop between insomnia, obesity, and stress disorders (15, 47–49), a more assertive approach to this component may also be important in managing NES, especially considering that improved sleep patterns positively impact the management of depressive disorders (50, 51), and also in managing overweight (52).

We also note the lack of evaluation of Topiramate in clinical trials, as this is a medication that may be used in cases of binge eating, having already been evaluated in sleep-related eating disorder (53), with therapeutic success.

Moreover, as guilt plays an important role as compensatory behavior in individuals with eating disorders (19), evaluating and addressing adequately such feeling could enhance treatment outcomes.

Further research should address such concerns adequately, with larger sample size, and possibly stratified subgroups regarding different severity guilt, anxiety, stress, insomnia and depressive symptoms, as this could reveal specific subgroups responses to different approaches, which may contribute to more tailored treatment approaches.

### 7.2 Quality of evidence

Despite including randomized controlled clinical trials with an overall low risk of bias, we downgraded the level of recommendation in GRADE for all outcomes to LOW. We made this decision due to the small number of participants in the studies, the fact that included studies utilized different interventions (only 2 trials evaluated applicability of sertraline), making a joint analysis impossible, as well as some risk of bias concerns (**Table 1**).

### 7.3 Implications for future research

This review highlights the need for further high-quality randomized controlled trials investigating both pharmacological and psychosocial interventions for Night Eating Syndrome (NES). The heterogeneity of existing studies, different interventions, small sample sizes, and limited follow-up periods hinder the generalizability of current findings. Future research should aim to standardize diagnostic criteria and outcome measures, explore combined treatment approaches, and assess long-term effects on symptom reduction, weight management, and quality of life. Additionally, studies should consider the role of comorbid psychiatric conditions and circadian rhythm disruptions in treatment response to better tailor interventions to patient subgroups.

In our findings, addressing adequately psychiatric comorbidities, such as guilt, stress, anxiety and insomnia showed consistent potential in controlling NES in our findings, including standardized measures of such emotional constructs may empower clinicians’ tools for improving outcomes. The use of combined strategies may also be useful, possibly stratified according to each individual pattern of psychosocial trait, and should be further evaluated as well.

## 8 Conclusion

Considering the complexity in managing eating compulsions in general, in light of the few quality studies already published, our findings suggest that a multidisciplinary approach may be beneficial for better control of NES. Perhaps the combination of psychotherapeutic approaches associated with pharmacological treatment targeting not only the general appetite control but also guilt, mood, metacognition and insomnia disorders may help improve symptom control.

Moreover, longer-term studies are warranted to evaluate long-term control of NES, as this is a chronic condition, and as such, there are progressive failures over time.

## Data Availability

The original contributions presented in the study are included in the article/supplementary material. Further inquiries can be directed to the corresponding author.
